# The relationship between stress and academic burnout in college students: evidence from longitudinal data on indirect effects

**DOI:** 10.3389/fpsyg.2025.1517920

**Published:** 2025-05-26

**Authors:** Jun Zhang, Jiawen Meng, Xin Wen

**Affiliations:** Department of Education, Sehan University, Yeongam-gun, Jeollanam-do, Republic of Korea

**Keywords:** stress, academic burnout, self-esteem, social support, indirect effects

## Abstract

**Objective:**

This study primarily examines the mechanisms through which stress affects academic burnout.

**Method:**

A total of 428 university students from three undergraduate institutions in China—Anhui Normal University, Tourism College of Zhejiang, and Bozhou University—were surveyed using the College Students' Stress Scale, the Academic Burnout Scale, the Social Support Scale, and the Self-Esteem Scale.

**Results:**

The results showed that stress significantly positively predicted academic burnout among college students and significantly negatively predicted their perceived social support. Both social support and self-esteem independently mediated the relationship between stress and academic burnout, and also served as a sequential (chain) mediator in this relationship.

**Conclusion:**

Stress can influence academic burnout both directly and indirectly. When individuals experience stress, their response to academic burnout is not only directly affected by the stress itself, but also indirectly influenced by two types of “psychological buffering resources”: internal resources (such as self-esteem) and external resources (such as social support). We refer to this phenomenon as the “Dual Buffering Path Model of Academic Burnout.” Based on these findings, it is necessary for educational authorities to take effective measures to reduce students' academic stress. In addition, friends, family, and teachers should offer emotional support, provide frequent positive feedback, and reinforce students' behaviors in order to foster their self-esteem and help them cope with academic burnout.

## 1 Introduction

Academic burnout refers to a negative emotional experience that students encounter during their learning process, characterized by a lack of enthusiasm, negative attitudes, and disengagement from academic tasks (Lei et al., 2022). This issue has become increasingly widespread among college students, exhibiting a high incidence and severity (Wang et al., 2021). However, specific manifestations of academic burnout can vary among students, highlighting its heterogeneity. Academic burnout not only affects academic performance and progress but also exacerbates mental health issues such as anxiety and depression, influencing personal career development and overall quality of life (Zhang L. et al., 2020). Research indicates a significant positive correlation between the academic stress faced by college students and their level of academic burnout: the greater the stress an individual faces, the higher the degree of academic burnout (Richardson et al., 2012). In recent years, the COVID-19 pandemic has had multifaceted impacts on students' academic experiences worldwide. First, the pandemic resulted in the temporary closure of schools and a shift from in-person to online learning. This sudden transition caused significant changes in the learning environment and methods, which may have decreased learning efficiency and increased academic stress (Wang et al., 2020). Second, students whose parents lost jobs due to COVID-19 may experience diminished academic performance, leading to increased academic burdens and feelings of anxiety (Brooks et al., 2020). Furthermore, social isolation during this period may exacerbate mental health issues, further affecting students' learning and concentration (Loades et al., 2020). Long-term academic burnout can lead to various adverse consequences, including psychological, physiological, and social issues. Psychologically, academic burnout may result in emotional problems such as depression and anxiety (Lim et al., 2016). Physiologically, it can impact sleep quality, leading to disorders and fatigue, thereby reducing individuals' life satisfaction and sense of wellbeing. Socially, academic burnout may impair social functioning, causing declines in social skills, intimate relationships, and interpersonal communication abilities. Therefore, investigating the mechanisms that lead to academic burnout among college students is essential. This understanding can help enhance learning efficiency and quality, reduce negative emotions, improve mental health levels, and boost individuals' self-confidence and self-efficacy.

Stress is a state of mental and physical tension that arises when individuals perceive stressors from internal or external sources. It typically manifests during the process of coping with challenges, demands, or difficulties, triggering a range of physiological and psychological responses (Zhang et al., 2024). Stress primarily affects three areas: cognition, emotion, and behavior (Smith and Johnson, 2020a). Cognitively, individuals may experience confusion and difficulty concentrating. Emotionally, they may exhibit anxiety, unease, and mood fluctuations. Behaviorally, stress can lead to withdrawal, impulsive actions, or negative coping strategies. If an individual remains in a state of stress for an extended period, it can result in issues such as anxiety, depression, physical discomfort, and poor sleep quality. These outcomes can potentially increase the risk of both psychological and physical illnesses, negatively impacting overall health (Zhang et al., 2024). A study on college students found a significant correlation between academic stress and academic burnout (Smith et al., 2020). When faced with academic stress, college students often experience a lack of motivation, low mood, and diminished interest in learning-all of which are associated with academic burnout. This situation has been exacerbated by the COVID-19 pandemic. During this time, students not only had to adapt to new learning environments and methods (Wang et al., 2020) but also faced feelings of loneliness and helplessness due to social isolation and economic difficulties at home. These factors contributed to increased psychological stress (Brooks et al., 2020; Loades et al., 2020) and the emergence of academic burnout.

Stress theory suggests that individuals may experience a range of physiological and psychological responses, such as anxiety, tension, and fatigue, when coping with academic stress. If these responses are not effectively alleviated or regulated, they can lead to the development of academic burnout. Conversely, academic burnout can exacerbate an individual's stress burden, creating a vicious cycle (Bakker et al., 2014). Some studies emphasize the importance of assessing the underlying mechanisms by which stress impacts academic burnout. This approach enhances our understanding of stress's influence on academic burnout and provides guidance for effective intervention measures (Zhang et al., 2019; Smith and Johnson, 2020b; Jones and Black, 2021). By exploring the relationship between stress and academic burnout in depth, researchers can identify potential indirect or moderating variables. This knowledge can help formulate targeted interventions that enhance college students' abilities to cope with stress and prevent academic burnout. Furthermore, understanding the deeper mechanisms by which stress affects academic burnout can assist in developing more effective prevention strategies. By identifying the sources of stress and the key factors related to academic burnout, schools and society can implement targeted mental health education and provide counseling services. Based on these insights, this study proposes Hypothesis 1: Stress can positively predict academic burnout.

### 1.1 The indirect role of social support

Social support refers to the resources individuals can access to alleviate stress or solve problems when facing life challenges or difficulties. This support can come from family, friends, colleagues, communities, and other social groups, positively impacting individuals' psychological and physical health (Holt-Lunstad et al., 2015). For individuals, social support manifests in feelings of being cared for, supported, and assisted by others during times of distress. This, in turn, reduces psychological burdens and stress while enhancing confidence and coping abilities. Academically, social support can be categorized into three dimensions: emotional support, tangible support, and informational support. These types of support typically originate from members of an individual's social network, such as family, friends, and colleagues. They can be expressed through emotional listening, understanding, providing practical help, or sharing information. Emotional support involves emotional understanding and resonance provided by others; tangible support refers to material or actionable assistance; and informational support encompasses advice or information that helps individuals solve problems. Research indicates that strong social support can alleviate stress, reduce the incidence of anxiety and depression, and promote overall psychological and physical wellbeing. Conversely, insufficient or negative social support can have detrimental effects (Uchino, 2009).

The impact of stress on social support is a complex process. Buffering theory posits that social support can mitigate the negative effects of stress from external environments on individuals (Uchino, 2009). This suggests that when individuals face stress, obtaining adequate social support can alleviate its adverse impacts and protect their mental health. Some studies indicate a negative correlation between stress and social support; in stressful situations, individuals often become more withdrawn, reducing their interactions with others and subsequently decreasing their social support (Zhang et al., 2024).

**Figure 1:**
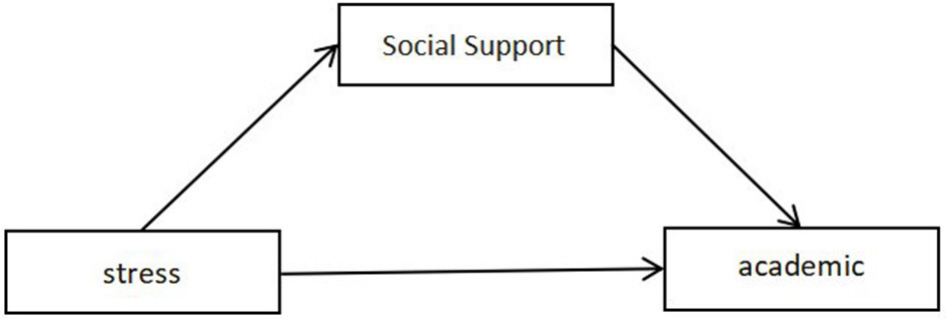
The indirect effect of social support between stress and academic burnout.

Social support theory further asserts that social support helps individuals cope with stress and adapt to life changes by providing emotional, informational, and tangible assistance (Holt-Lunstad et al., 2015). Research has identified a significant negative correlation between social support and academic burnout: the more abundant the social support, the lower the levels of academic burnout, which in turn enhances students' academic performance and mental health (Hu, 2025). These findings emphasize the crucial role of social support in alleviating academic burnout and suggest that fostering a healthy social support network is beneficial for students in managing academic stress and maintaining motivation. Consequently, this study proposes Hypothesis 2: social support plays an indirect role in the relationship between stress and academic burnout, as illustrated in the model in Figure 1.

### 1.2 The indirect role of self-esteem

Self-esteem refers to an individual's perception and evaluation of themselves, including feelings and views about their abilities, worth, and identity. It reflects one's attitude and emotions toward oneself (Mruk, 2019; Baumeister et al., 2019). The structure of self-esteem is generally divided into two aspects: positive self-esteem and negative self-esteem. Positive self-esteem involves favorable recognition and evaluation of one's abilities and value, whereas negative self-esteem includes unfavorable perceptions and evaluations of oneself (Orth and Robins, 2014). Research has shown that high levels of self-esteem are associated with better mental health, more positive social relationships, and greater life satisfaction, while low levels of self-esteem may lead to emotional problems, social difficulties, and mental health issues, potentially affecting an individual's quality of life and life satisfaction (Kernis, 2018). Additionally, self-esteem is closely related to social support. Having strong social support can enhance self-esteem, thereby promoting mental health and social adaptability (Orth and Robins, 2014).

According to the self-evaluation theory, an individual's level of self-esteem is shaped by comparisons with others. When such comparisons generate negative emotions, the resulting stress may cause the individual to feel insecure, helpless, and inadequate, potentially lowering their self-esteem (Buunk and Gibbons, 2017; Sowislo and Orth, 2013). Studies have found a negative correlation between stress and self-esteem; students experiencing academic stress often report more problems and difficulties related to self-esteem (Yan, 2023).

**Figure 2:**
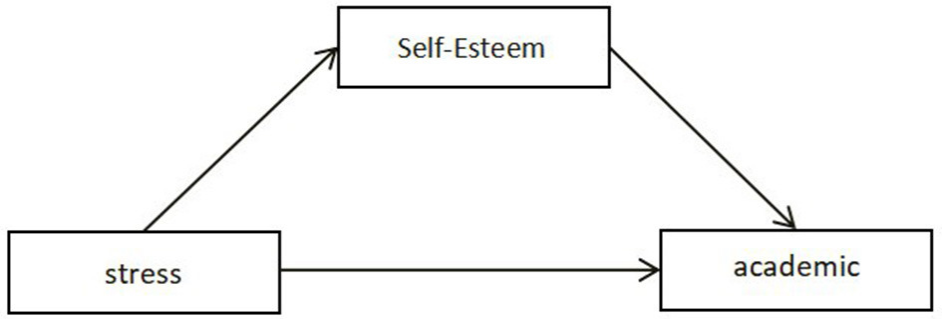
The indirect effect of self-esteem between stress and academic burnout.

The impact of self-esteem on academic burnout is a key topic in psychological research. Social cognitive theory posits that an individual's self-esteem influences their attitude and confidence toward academic tasks. Those with lower self-esteem are more likely to be affected by setbacks and failures, resulting in academic burnout. Conversely, individuals with high self-esteem may be more motivated to face challenges and difficulties, thus demonstrating greater academic engagement and enthusiasm (Pekrun et al., 2017). One study found a negative correlation between students' self-esteem and academic burnout, indicating that lower self-esteem is associated with higher levels of burnout. Moreover, self-esteem is positively correlated with academic performance and engagement; the higher the self-esteem, the better the academic outcomes and involvement (Liu Y., 2023). Based on this, the current study proposes Hypothesis 3: Self-esteem plays a mediating role in the relationship between stress and academic burnout. The hypothesized model is shown in Figure 2.

### 1.3 The chain of indirect effects of social support and self-esteem

**Figure 3:**
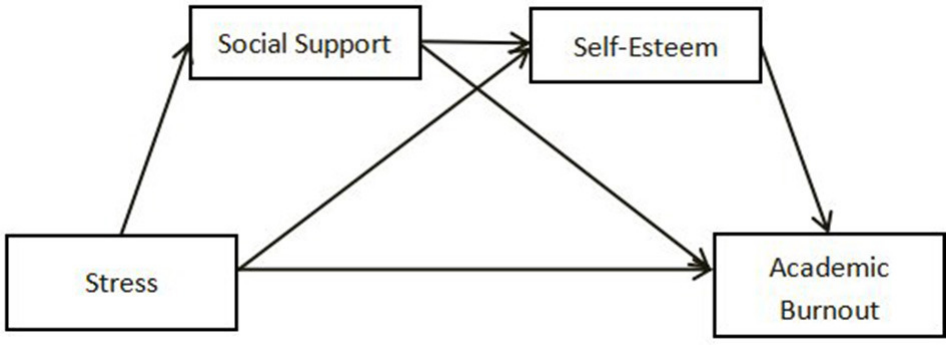
The indirect effects of social support and self-esteem between stress and academic burnout.

Social support can be defined as the extent to which individuals perceive emotional, instrumental, and informational assistance from others, such as family, friends, and colleagues. This support provides not only emotional comfort and encouragement but also resources and information that can enhance self-confidence. Research has found a positive correlation between social support and self-esteem, indicating that perceived family support is closely linked to higher levels of self-confidence. In other words, individuals who perceive greater family support tend to have higher self-esteem (Hu, 2025). These findings underscore the significant impact of social support on self-confidence, suggesting that enhancing social support can lead to improved levels of self-esteem. Based on this understanding, the present study proposes Hypothesis 4: Social support and self-esteem play a chain of indirect roles in the relationship between stress and academic burnout, as illustrated in Figure 3.

## 2 Method

### 2.1 Participants

A cluster sampling method was employed to select participants from 30 universities and colleges in Wuhu City and Bozhou City of Anhui Province, and Hangzhou City of Zhejiang Province, China. Ultimately, students from three universities—Anhui Normal University, Tourism College of Zhejiang, and Bozhou University—were selected to participate in the study. The sample included all enrolled students from the first, second, and third years. Fourth-year students were excluded from the study due to their off-campus internships. We strictly adhered to the relevant guidelines of the Declaration of Helsinki. The study officially commenced only after receiving approval from the ethics committee responsible for reviewing this research. Since this was a longitudinal study with data collection conducted at 3-month intervals, the first round of testing was carried out on May 16, 2023, during which 437 questionnaires were distributed and collected; stress, as the independent variable, was measured in this round. The second round took place on August 19, 2023, with 442 questionnaires distributed and collected; data on the mediating variables, social support and self-esteem, were gathered during this phase. The third round occurred on November 21, 2023, with 433 questionnaires distributed and collected; academic burnout, as the dependent variable, was measured. Before the formal survey, we explained the purpose and procedures of the study to all participants. Informed consent was obtained from each participant after ensuring that they fully understood the study. The formal survey was conducted in classrooms, where participants independently completed demographic questions and questionnaires on stress, academic burnout, self-esteem, and social support. As this was a longitudinal study, we included only those participants who completed all three rounds of testing. Questionnaires from participants who were absent from any of the tests due to illness, leave, or other reasons were excluded. After removing invalid responses, a total of 428 valid questionnaires were retained. Among the valid participants, 109 (25.5%) were male and 319 (74.5%) were female; 347 (81.1%) came from rural areas, and 81 (18.9%) were from urban areas.

### 2.2 Research tools

#### 2.2.1 College student stress scale

The College Student Stress Scale, developed by Li and Mei (2002), was used to assess the stress levels of college students. This scale comprises three factors: academic distress, personal distress, and negative life events, totaling 30 items. It employs a 4-point Likert scoring system, where participants rate their responses from 0 (no stress) to 3 (severe stress). A higher score indicates greater perceived stress at that moment (Zhang et al., 2024). In this study, the Cronbach's alpha coefficient for the scale was 0.96, indicating excellent internal consistency.

#### 2.2.2 Academic burnout scale

The Academic Burnout Scale for adolescents, developed by Wu et al. (2010), was used to measure learning burnout among college students. This scale consists of 16 items that assess three dimensions: emotional exhaustion (e.g., “I feel very empty lately and don't know what to do”), academic alienation (e.g., “I feel that it doesn't matter whether I study or not”), and low achievement (e.g., “When studying, I forget everything around me”). Participants rated their responses using a 5-point Likert scale, with options ranging from 1 (not at all true) to 5 (very true). A higher total score indicates greater severity of academic burnout experienced by the participants (Wu et al., 2010). Due to the heterogeneity observed in individual responses to the Academic Burnout Scale in Study 1—particularly concerning low achievement across the three groups—this study focused only on the dimensions of emotional exhaustion and academic alienation. The Cronbach's alpha coefficient for the scales incorporating these two dimensions was 0.89, indicating good internal consistency.

#### 2.2.3 Social support scale

The Social Support Scale developed by Xiao Shuiyuan was utilized in this study. This scale includes three dimensions: objective support (e.g., “In the past, what sources of financial support and practical help did you receive during emergencies?”), subjective support (e.g., “How many close friends can you rely on for support and help?”), and the utilization of social support (e.g., “What methods do you use to seek help when you encounter distress?”). It comprises a total of 10 items, with 3 items in the objective support dimension, 4 items in the subjective support dimension, and 3 items related to the utilization of social support.

For items 1–4 and 8–10, participants select one option, scored from 1 to 4 points. Item 5 offers five options (A, B, C, D, E) with scores ranging from “none” to “full support,” recorded as 1–4 points (1 point for “none,” 2 points for “very little,” 3 points for “moderate,” and 4 points for “full support”). For items 6 and 7, if participants answer “no sources,” they receive 0 points; if they indicate sources, they receive points equal to the number of sources mentioned (Jia et al., 2023). The Cronbach's alpha coefficient for this scale in this study was 0.97, indicating acceptable reliability.

#### 2.2.4 Self-esteem scale

The Self-Esteem Scale developed by Rosenberg (1965) was utilized in this study. This scale consists of a single dimension with a total of 10 items, including 5 positively worded items (e.g., “I feel that I am a person of worth, at least on par with others”) and 5 negatively worded items (e.g., “Ultimately, I tend to feel that I am a failure”). A 4-point Likert scoring method was employed, where participants selected options ranging from 1 (very untrue) to 4 (very true). The total score on the scale ranges from 10 to 40, with higher scores indicating greater levels of self-esteem (Liu et al., 2017). In this study, the Cronbach's alpha coefficient for this scale was 0.78, reflecting acceptable reliability.

### 2.3 Research procedure

We utilized SPSS 25.0 software to analyze the means, standard deviations, and Pearson correlation coefficients of stress, academic burnout, social support, and self-esteem. Additionally, we conducted confirmatory factor analysis using Mplus 7.0 to assess the internal structural validity of the questionnaires, ensuring that there were no missing data. After confirming that the structural validity indicators for the stress, academic burnout, social support, and self-esteem questionnaires fit reasonably, we constructed a structural equation model to verify the validity of the chain of indirect effects model. Our research proceeded in two steps. In the first step, we treated stress as the independent variable and academic burnout as the dependent variable to test for a significant direct effect of stress on academic burnout. In the second step, we examined the independent indirect effects and the chain of indirect effects of social support and self-esteem between stress and academic burnout, with stress as the independent variable and academic burnout as the dependent variable.

For evaluating the fit of the structural model, we used the fit indices proposed by Wen et al. (2004) and Brown (2015). Specifically, the model is considered to fit well if RMSEA <0.1, SRMR <0.1, TLI > 0.9, and CFI > 0.9 (Zhang et al., 2024; Brown, 2015).

## 3 Results

### 3.1 Control and testing for common method bias

This study employed various measurement tools, all utilizing self-report formats to collect data. Consequently, it is essential to verify the presence of common method bias. We conducted a factor analysis using Harman's single-factor method, which extracted a total of 11 factors with eigenvalues >1, explaining 67.13% of the total variance. The first principal factor accounted for 32.08% of the variance, which is below the critical threshold of 40% (Zhang et al., 2024). This finding indicates that common method bias is not an issue in this study.

### 3.2 Descriptive statistics and correlation analysis

We used independent samples *t*-tests to examine the differences in demographic variables, including household registration and gender, with respect to stress, academic burnout, social support, and self-esteem. The analysis revealed no significant differences between boys and girls in terms of academic burnout (*t* = −1.22, *p* > 0.05), social support (*t* = −1.23, *p* > 0.05), and self-esteem (*t* = 0.44, *p* > 0.05). However, a significant difference was found in stress scores (*t* = −2.13, *p* < 0.05).

When comparing urban and rural college students, there were no significant differences in stress (*t* = 0.63, *p* > 0.05), academic burnout (*t* = 0.43, *p* > 0.05), social support (*t* = −0.76, *p* > 0.05), or self-esteem (*t* = −1.52, *p* > 0.05), as shown in Table 1.

**Table 1:** Differences in stress, academic burnout, social support, and self-esteem among university students by gender and household registration.

**Dependent variable**	**Independent variable**	***F***	**Sig**	***t***	**Sig (two-tailed)**
Stress	Sex	8.63	0.00	−2.13	0.03
Academic burnout		0.27	0.60	−1.22	0.22
Social support		2.00	0.15	−1.23	0.21
Self-esteem		8.53	0.00	0.44	0.65
Stress	Household registration	6.37	0.01	0.63	0.52
Academic burnout		0.03	0.85	0.43	0.66
Social support		2.91	0.08	−0.76	0.44
Self-esteem		0.07	0.78	−1.52	0.12

We also employed one-way ANOVA to analyze differences in stress, academic burnout, social support, and self-esteem across different grade levels. This analysis revealed significant differences in stress scores among students of varying grades (*F* = 3.99, *p* < 0.05), but no significant differences were observed in academic burnout (*F* = 0.09, *p* > 0.05), social support (*F* = 1.54, *p* > 0.05), or self-esteem (*F* = 0.69, *p* > 0.05), as detailed in Table 2. Finally, the means, standard deviations, and correlation matrices for each variable are presented in Table 3.

**Table 2:** Differences in stress, academic burnout, social support, and self-esteem among college students of different grades.

**Dependent variable**	**Source of variation**	**Independent variable**	**Sum of squares**	**Degrees of freedom**	**Mean square**	***F***	**Sig (two-tailed)**
Stress	Inter-group	Grade	1,752.00	2	876.00	3.99	0.01
	Intra-group		93,240.67	425	219.39		
Academic burnout	Inter-group		14.65	2	7.32	0.09	0.90
	Intra-group		31,471.18	425	74.05		
Social support	Inter-group		186.54	2	93.27	1.54	0.21
	Intra-group		25,620.17	425	60.28		
Self-esteem	Inter-group		23.10	2	11.55	0.69	0.50
	Intra-group		7,089.35	425	16.68		

**Table 3:** Mean, standard deviation, and correlation coefficients of variables.

**Variable**	**1**	**2**	**3**	**4**	**5**	**6**	**7**
1. Sex	1						
2. Grade	0.00	1					
3. Household registration	0.02	−0.05	1				
4. Stress	0.10^*^	0.12^*^	−0.03	1			
5. Academic burnout	0.13^**^	0.04	−0.05	0.55^**^	1		
6. Social support	0.06	0.03	0.03	−0.26^**^	−0.30^**^	1	
7. Self-esteem	−0.02	−0.05	0.07	−0.45^**^	−0.49^**^	0.40^**^	1
*M*	1.75	1.13	1.19	23.60	22.26	36.52	27.92
SD	0.43	0.42	0.39	14.91	6.59	7.77	4.08

N = 428.

^*^p < 0.05, ^**^p < 0.01.

### 3.3 Construction and testing of the structural equation model

**Figure 4:**
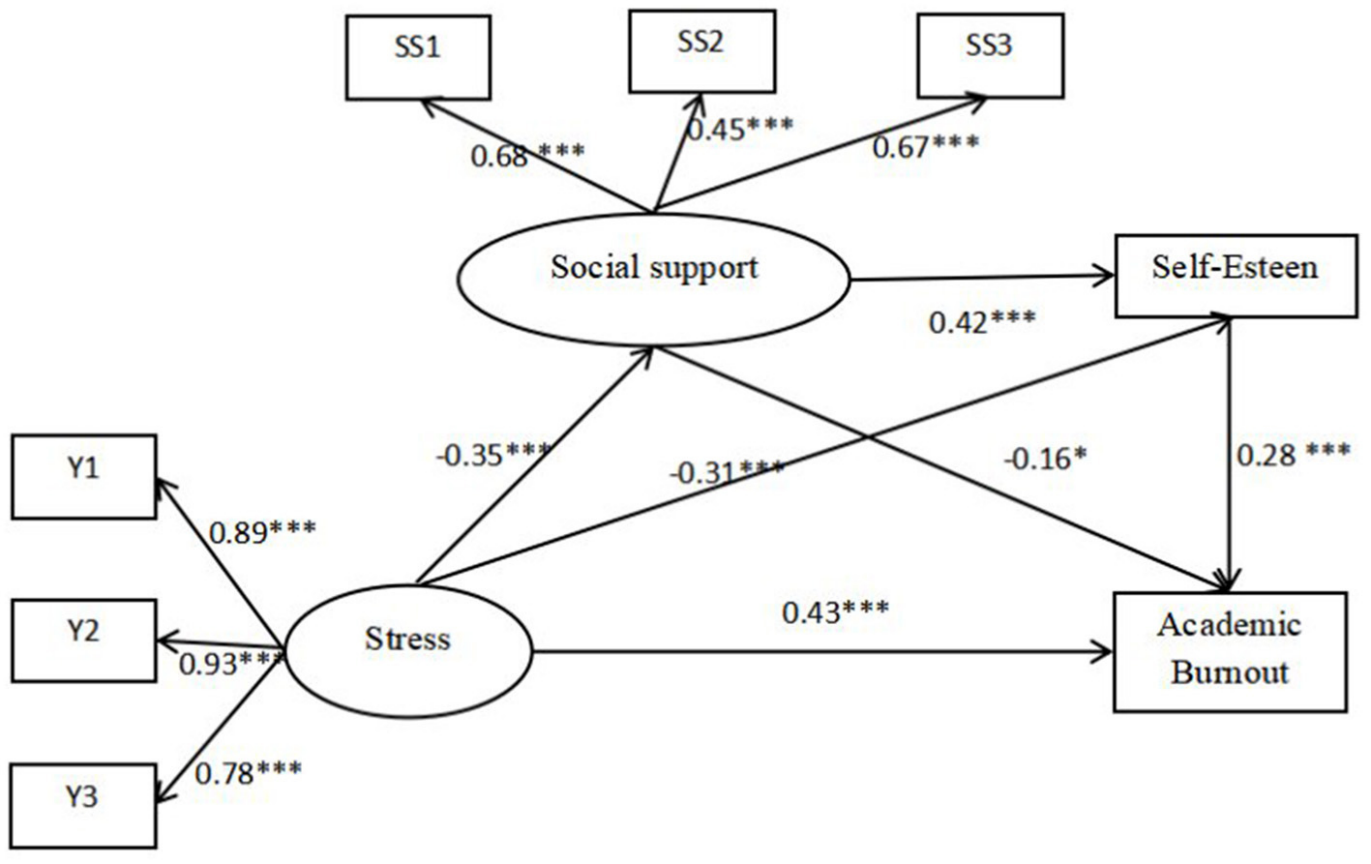
The relationship between stress and academic burnout: the indirect effects of social support and self-esteem. ^*^p <0.05, ^**^p <0.01, ^***^p <0.001.

In this study, we utilized the stress scale, social support scale, and academic burnout scale, each comprising a substantial number of items. Direct modeling could potentially compromise the quality of the indicator data and the true structure of the model. To address this, we employed an item-packaging method for modeling (Zhang et al., 2024). Additionally, we included gender, age, and household registration as control variables in our analysis, as these factors were significantly correlated with other study variables. Firstly, we tested whether stress could directly predict academic burnout. The results indicated that stress significantly and positively predicted academic burnout (β = 0.50, *p* < 0.001), with good model fit indices: RMSEA = 0.00, SRMR = 0.00, TLI = 1.00, CFI = 1.00. Next, we constructed a structural equation model with stress as the independent variable, social support and self-esteem as indirect variables, and academic burnout as the dependent variable. This model also demonstrated good fit indices: RMSEA = 0.06, SRMR = 0.04, TLI = 0.94, CFI = 0.95. In the indirect effects model, stress positively predicted academic burnout (β = 0.43, *p* < 0.001) and negatively predicted both social support (β = −0.35, *p* < 0.001) and self-esteem (β = −0.31, *p* < 0.001). Conversely, social support positively predicted self-esteem (β = 0.42, *p* < 0.001) and negatively predicted academic burnout (β = −0.16, *p* < 0.05). Notably, self-esteem also positively predicted academic burnout (β = 0.28, *p* < 0.001). For detailed results, see Figure 4.

We employed bootstrap resampling with 1,000 iterations to test the chain of indirect effects, using a 95% confidence interval. The results indicated that both social support and self-esteem had significant indirect effects between stress and academic burnout, with a total indirect effect of 0.06. Specifically, the pathway effects were as follows: stress → social support → academic burnout (0.02 [0.00–0.05]), stress → self-esteem → academic burnout (0.03 [0.01–0.05]), and stress → social support → self-esteem → academic burnout (0.01 [0.00–0.03]). Notably, all three indirect pathways had bootstrap 95% confidence intervals that did not include zero, confirming the significance of these effects. Therefore, hypotheses 1, 2, 3, and 4 of this study are supported, as detailed in Table 4.

**Table 4:** Bootstrap analysis of significance testing for indirect effects.

**Intermediary path**	**Effect size**	**95% confidence interval**	
		**Lower limit**	**Upper limit**
Stress → Social Support → Academic Burnout	0.02	0.00	0.05
Stress → Self-Esteem → Academic Burnout	0.03	0.01	0.05
Stress → Social Support → Self-Esteem → Academic Burnout	0.01	0.00	0.03

## 4 Discussion

This study found that stress significantly predicts academic burnout, thereby validating Hypothesis 1. The stress-adaptation model suggests that an individual's adaptation process consists of three stages: coping and regulation, restoring balance, and learning and growth. During the coping and regulation stage, individuals employ various strategies to manage stress. In the restoring balance stage, they seek to regain internal and external equilibrium. Finally, in the learning and growth stage, individuals enhance their coping abilities through experience and reflection, better preparing themselves for future challenges (McEwen, 2017). Following the outbreak of the COVID-19 pandemic, many families faced numerous stressors, including health panic, social isolation, economic uncertainty, job loss, strained family relationships, and information overload (Brooks et al., 2020; Rajkumar, 2020; Shigemura et al., 2020). The stress experienced by individuals can transfer to family members, resulting in increased tension, conflict, and emotional volatility within the household (Prime et al., 2020; Spinelli et al., 2020; Taubman-Ben-Ari et al., 2020). For students, prolonged isolation has led to heightened academic pressures, cancellations or delays of exams, lack of face-to-face interactions with classmates, inadequate learning resources, and unfavorable home environments (Cao et al., 2020; Elmer et al., 2020; Zhang et al., 2020a). Therefore, it is crucial to examine the relationship between academic burnout, the sources of stress, and coping strategies. Stress can disrupt cognitive functions and learning processes, resulting in difficulties in learning and decreased efficiency, which ultimately contribute to academic burnout (Kong et al., 2019). Additionally, emotional and psychological responses to stress in educational contexts—such as negative emotions and anxiety—can impact students' motivation and engagement, leading to a loss of interest in their studies. This, in turn, results in negative academic behaviors and avoidance, further contributing to academic burnout (Zhang et al., 2020b). In China, despite educational authorities advocating for quality education, exam results remain the primary criterion for evaluating students. The pressure from schools and parents often leads to various forms of extracurricular learning, overwhelming many students with stress and prompting some to drop out (Pan, 2014). Our study confirms that increased stress levels positively predict academic burnout, indicating that rising stress can negatively affect children's interest in learning. This underscores the need for Chinese educational institutions and families to address this issue by appropriately reducing academic stress on children.

This study confirmed the indirect effect of social support between stress and academic burnout, validating Hypothesis 2. Social support theory posits that support from social networks and interpersonal relationships can significantly enhance an individual's psychological and physiological health when facing stress and challenges. This support encompasses emotional assistance (such as comfort and understanding), informational guidance (such as advice and resources), and tangible help (such as material support). The theory underscores the importance of interpersonal relationships in fostering individual adaptability and coping skills, suggesting that those with robust social support systems are better equipped to manage life's stressors (Holt-Lunstad et al., 2017). Social support can alleviate stress, improve mental health, enhance coping abilities, and promote psychological adjustment (Cohen and Janicki-Deverts, 2012; Uchino, 2009). Internally, it provides emotional backing and understanding, which reduces negative emotions and anxiety while boosting self-esteem and confidence (Zhen et al., 2014). Externally, social support facilitates access to information and resources, enabling individuals to effectively tackle academic challenges, thereby mitigating academic stress and reducing the risk of academic burnout (Liu et al., 2020). However, research indicates that an increasing number of Chinese students are becoming addicted to video games, leading to greater social isolation (Zhang and Wang, 2010). This trend negatively impacts their levels of social support. Consequently, it is essential for schools and families to emphasize the dangers of excessive video game use and to limit the time children spend playing them.

This study confirmed the indirect role of self-esteem in the relationship between stress and academic burnout, thereby validating Hypothesis 3. Self-esteem theory suggests that an individual's self-evaluation influences their engagement and effort in academic pursuits. Higher self-esteem boosts confidence and motivation toward learning, making individuals more willing to face challenges and maintain a positive attitude, ultimately reducing the likelihood of academic burnout. Conversely, lower self-esteem can lead to self-doubt, diminishing engagement and motivation in academic settings, which increases the risk of burnout (Orth and Robins, 2013). The development of self-esteem is shaped by both internal factors—such as cognitive, emotional, and behavioral aspects—and external influences, including social environments and others' evaluations (Orth and Robins, 2014). While self-esteem levels can fluctuate based on individual experiences and changes in social contexts, they tend to remain relatively stable and exert a lasting impact on resilience and confidence (Miao et al., 2020). High self-esteem correlates positively with psychological wellbeing, adaptability, and lower levels of anxiety and depression, as well as with positive social interactions, greater life satisfaction, and happiness (Orth and Robins, 2014). Individuals with high self-esteem are generally better equipped to cope with stress, effectively reducing negative emotions and anxiety (Chang et al., 2019). They are also more likely to establish positive goals and coping strategies, enabling them to tackle academic challenges, alleviate stress, and reduce the risk of burnout (Zhou et al., 2021). In China, many parents feel anxious about their children's academic performance (Meng, 2021) and often seek to improve grades through after-school tutoring. However, this approach can yield mixed results (Liu and Wang, 2018). If parents and teachers focus on reducing academic stress during the educational process and emphasize fostering self-esteem alongside encouraging independent learning, it could lead to more beneficial outcomes for students.

This study found that social support and self-esteem play a chain-mediating role in the relationship between stress and academic burnout, thus supporting Hypothesis 4. According to the buffering hypothesis of social support, social support can serve as a “buffer” when individuals encounter stress. Such positive experiences of support help individuals gain a sense of value and wellbeing, thereby enhancing their self-esteem (Cohen and Wills, 1985; Marshall et al., 2014). Individuals with high levels of self-esteem tend to exhibit stronger self-efficacy and greater enthusiasm for learning, which in turn reduces levels of academic burnout (Liu T., 2023). Social support can offer emotional validation and attachment, provide information and feedback, and alleviate psychological burdens when individuals face challenges and stress, thus promoting and maintaining a sense of self-worth, reducing academic burnout, and supporting students' academic achievement and mental health (Ginns et al., 2021; Reevy and Deason, 2014). Unlike previous studies, this research found that self-esteem could positively predict academic burnout, which may be attributed to unstable self-esteem. Unstable self-esteem can impair individuals' psychological adaptability to stress, thereby contributing to academic burnout (Zeigler-Hill and Wallace, 2012; Gao, 2014). These findings suggest that the mechanism by which stress affects academic burnout is complex. To improve students' academic burnout levels, we should address the root causes by reforming current educational policies—academic performance should not be the sole criterion for admission to prestigious schools but rather one component of a comprehensive evaluation system. Additionally, both schools and families should proactively reduce academic pressure, avoid assigning extra-curricular tutoring beyond regular schoolwork, encourage students to engage more in social interactions, and work on enhancing their self-esteem, ultimately helping to alleviate academic burnout.

## 5 Limitations and implications

We acknowledge several limitations in this study. First, our sample size of 428 participants is relatively small, which may affect the generalizability of our findings to other populations. Second, although we conducted three waves of longitudinal data collection, we chose a three-month interval based on the practices of other researchers. However, this interval may not adequately capture changes in psychological states and might not fully reflect the actual circumstances of the participants.

Despite these limitations, this research supports the theories of self-esteem, social support, and the stress-adaptation model, while also applying these theories in empirical contexts. Furthermore, the use of longitudinal data enhances the credibility of our findings. By constructing a structural equation model, we explored the mechanisms through which stress influences academic burnout, contributing valuable insights to the theoretical and empirical understanding of the relationship between these two factors.

## 6 Conclusion

Stress can both directly and indirectly predict academic burnout. Academic burnout is not only directly influenced by stress but also indirectly affected by two types of “psychological buffering resources”—internal resources (such as self-esteem) and external resources (such as social support). We refer to this phenomenon as the “Dual-Buffering Pathway Model of Academic Burnout.” In China, schools and parents have long placed a strong emphasis on students' academic performance, often setting overly high expectations during the educational process. This immense pressure may contribute to heightened academic burnout among students. Therefore, both school and family education in Chinese society should pay greater attention to students' inner thoughts, abandon the practice of using academic performance as the sole evaluation criterion, and focus on the holistic development of students' abilities. Encouraging children to develop their strengths based on their interests, and providing them with consistent affirmation, support, and understanding during their growth process, can be effective in alleviating academic burnout.

## Data Availability

The raw data supporting the conclusions of this article will be made available by the authors, without undue reservation.
